# Resting heart rate and the risk of incident type 2 diabetes mellitus among non-diabetic and prediabetic Iranian adults: Tehran lipid and glucose study

**DOI:** 10.1186/s12889-023-17022-7

**Published:** 2023-10-27

**Authors:** Seyyed Saeed Moazzeni, Kimia Karimi Toudeshki, Fatemeh Ghorbanpouryami, Mitra Hasheminia, Fereidoun Azizi, Mehdi Pishgahi, Farzad Hadaegh

**Affiliations:** 1 Prevention of Metabolic Disorders Research Center, Research Institute for Endocrine Sciences, Shahid Beheshti University of Medical Sciences, Tehran, Iran; 2 Medical student, Faculty of Medicine, Shahid Beheshti University of Medical Sciences, Tehran, Iran; 3 Endocrine Research Center, Research Institute for Endocrine Sciences, Shahid Beheshti University of Medical Sciences, Tehran, Iran; 4 Department of Cardiology, Shohada-e-Tajrish Hospital, Shahid Beheshti University of Medical Sciences, Tehran, Iran

**Keywords:** Heart rate, Diabetes Mellitus Type 2, Prediabetic state, Iran

## Abstract

**Background:**

Resting heart rate (RHR) has been found to be a potential risk factor for developing type 2 diabetes mellitus (T2DM), with a highly significant heterogeneity among previous studies. Therefore, we examined the association of RHR and risk of incident T2DM among non-diabetic and prediabetic adults.

**Methods:**

The study population included 2431 men and 2910 women aged ≥ 20 years without T2DM at baseline (2001–2005). Participants were followed for incident T2DM by about 3-year intervals up to April 2018. The multivariable Cox proportional models were applied to estimate the hazard ratios (HRs) and 95% confidence intervals (CIs). The models were adjusted for age, body mass index, waist circumference, educational level, physical activity, smoking, hypertension, family history of diabetes, triglycerides/ high-density lipoprotein cholesterol ratio, and fasting plasma glucose.

**Results:**

During a median follow-up of 12.2 years, 313 men and 375 women developed T2DM. Interestingly, a significant sex-difference was found (all P-values for sex interaction < 0.025). Among men, compared to the first quintile (< 68 bpm: beats per minute), those who had RHR of over 84 bpm were at higher T2DM risk with a HR (95%CI) of 1.69 (1.16–2.47). Furthermore, considering RHR as a continuous variable, an increase of 10 bpm caused 17% significantly higher risk among men with a HR of 1.17 (1.05–1.30). However, among women, there was no significant association between incident T2DM and RHR. Moreover, among prediabetic participants at baseline, the association of RHR and risk of T2DM progression was generally similar to the general population, which means higher RHR increased the risk of T2DM development only among men with a HR of 1.26 (1.09–1.46) for 10 bpm increase.

**Conclusions:**

Among men, being either non-diabetic or prediabetic at baseline, higher RHR can be associated with incident T2DM; however, women didn’t show a significant association. Further studies are needed to determine the added value of RHR as a potential modifiable risk factor in screening and risk prediction of incident T2DM.

## Background

Type 2 diabetes mellitus (T2DM) caused approximately 1.5 million deaths and was the 8th leading cause of disability-adjusted life years (DALYs) in 2019 globally [1]. In 2021, the Middle East and North Africa region (MENA) had the highest standardized prevalence of T2DM globally (18.1%), with an increasing trend [2]. National data from Iran demonstrated that 15.0% and 25.4% of Iranian adults had diabetes and prediabetes, respectively [3]. Moreover, we previously found an age standardized incidence rate of 9.94 per 1000 person-years for T2DM among adults residents of Tehran [4, 5]. A prediabetes tsunami was also reported among residents of Tehran, with > 4% of the population developed prediabetes annually [6].

Besides well-known T2DM risk factors, including obesity, physical inactivity, dietary factors, and genetic susceptibility [7], resting heart rate (RHR) has been shown to be significantly associated with incident T2DM and prediabetes [8–11]. This association has been suggested to be primarily attributed to the insulin resistance (IR) mediated by sympathetic/parasympathetic system [8]. Two previous meta-analyses showed that an increase of 10 beats per minute (bpm) was accompanied with approximately 20% higher T2DM risk; a similar significant higher risk was also reported for high RHR categories versus the lowest categories [8, 10]. However, results from these two meta-analyses had a highly significant heterogeneity (all I^2^ were about 90%) [8, 10]; the relationship between RHR and incident T2DM was more prominent among Asian populations, compared to Western ones [8]. As far as we know, there is no previous study that examined the corresponding relationship in the MENA region.

Therefore, the current study has the aim of investigation of sex-specific relationship between T2DM development and RHR (assessed through palpation) among non-diabetic and prediabetic Iranian adults, using data from the oldest cohort in the MENA region.

## Methods

### Study design and population

The Tehran Lipid and Glucose Study (TLGS), conducting since 1999, is a prospective population-based study about epidemiological features of non-communicable diseases (NCDs). Data from more than 15,000 Tehranian residents of district 13 has been collected during the recruitment phases. Then it was planned to follow participants by about 3-year intervals [12]. The TLGS aims to make prevention of NCDs through a healthier lifestyle [12]. For the current study, the second phase of TLGS (October 20, 2001 to September 22, 2005) was considered as enrollment. Data gathering for follow-up was carried out in phase III (2005–2008), phase IV (2008–2011), phase V (2011–2014), and phase VI (2015–2018). Further details about TLGS rational and design were described before [12].

From a total of 3891 men and 5036 women aged > 20 years, 443 men and 623 women were excluded due to having T2DM at baseline. Furthermore, we excluded 213 men and 173 women with prevalent cardiovascular disease (CVD) or cancer at baseline. Another exclusion reason was using antihypertensive or vasodilator medications at baseline (255 men and 612 women), leading to 2980 men and 3628 women. Then we excluded 318 men and 442 women, due to baseline missing data on RHR, fasting plasma glucose (FPG), 2-h post-challenge plasma glucose (2 h PG), and related covariates (considering overlap features). Finally, after further exclusion of 231 men and 276 women because of no follow up data, 2431 men and 2910 women were eligible to enter the analysis.

### Clinical and laboratory measurement

Using standard questionnaires, data on age, educational level, smoking habits, physical activity, as well as medical records (history of major illnesses, medication usage, family history of cardiovascular disease and diabetes) obtained. Weight and height were measured while participants were wearing light clothes and in a standing position. Body mass index (BMI) was calculated as weight [kg]/ (height [m])^2^. We also measured waist circumference (WC) at the level of the umbilicus with light clothing. Additionally, after 15 min of rest, blood pressure (BP) was assessed twice in a seated position, using manual sphygmomanometer. Through radial palpation, RHR was assessed twice and counted over 60-s periods. The mean of two numbers was considered as pulse rate.

All study participants were asked to fast on the day of the visit for at least 12 h before blood sampling. From all individuals, blood samples were taken with a standard method and in a sitting position. To assess 2 h PG, 82.5 g glucose monohydrate solution (equivalent to 75 g anhydrous glucose) was orally taken by individuals who had not a history of taking any glucose-lowering medications. Further details on standard methods for the measurement of 2 h PG, FPG, high-density lipoprotein cholesterol (HDL-C), and triglycerides (TG) were expressed elsewhere [12].

### Definition of terms

In this study, the level of education was sorted into 3 levels of ≤ 6, 6 to 12, and over 12 years of formal education. Subjects were also classified in two groups of current smokers versus previous/non-smokers. Hypertension was considered systolic blood pressure (SBP) ≥ 140 mmHg, or diastolic blood pressure (DBP) ≥ 90 mmHg, or taking anti-hypertensive drugs [13].

Moreover, using the Modifiable Activity Questionnaire (MAQ), individuals who had less than 1500 min per week of metabolic equivalent tasks were considered as the low physical activity group [12, 14].

T2DM was considered as using glucose lowering drugs (GLDs), or FPG of ≥ 7 mmol/L, or 2 h PG ≥ 11.1 mmol/L. Prediabetes was also considered as 7 mmol/L > FPG ≥ 5.6 mmol/L, or 11.1 mmol/L > 2 h PG ≥ 7.7 mmol/L [15].

Homeostasis Model Assessment of Insulin Resistance (HOMA-IR) was determined using the formula: FPG(mmol/L)*fasting insulin(Mu/ml)/22.5 [16].

### Statistical analysis

Baseline demographic and clinical characteristics across the quintiles of RHR are displayed as mean ± standard deviation (SD), median (interquartile range: IQR), and number (%) for normally distributed continuous, highly skewed, and categorical variables, respectively. Chi-square and ANOVA tests were employed to compare baseline characteristics among different groups.

Time-to-event was considered as the time of event occurring or censoring, whichever came first. Participants were censored if they died during the follow-up, left the district, or did not develop T2DM until the end of phase VI (April 2018). Survival time for the censored individuals was the interval between the first and the last observation dates. The event date for the cases of incident T2DM was considered as the mid-time between the date of follow-up visit at which T2DM was detected for the first time, and the most recent follow-up visit preceding the diagnosis.

The Cox proportional hazard models were employed to assess the association of RHR with incident T2DM by calculating the sex-specific hazard ratios (HRs) with 95% confidence intervals (CIs). Four models were used in this analysis: Model 1 adjusted for age. Model 2 adjusted for traditional T2DM risk factors including age, WC, BMI, educational level, current smoking, low physical activity, family history of diabetes, and hypertension. Model 3: Model 2 + further adjusted for TG/HDL-C ratio; Model 4: Model 3 + further adjusted for FPG. For categorization of RHR, the main exposure of this study, we considered 5 quintiles for each gender separately (the lowest quintile as reference). Considering RHR as a continuous variable, the effect sizes were also calculated for an increase of 10 bpm. As a sensitivity analysis, we also adjusted our models with HOMA-IR for the subgroup of 1394 men and 1849 women with available date for baseline fasting insulin. Due to a significant interaction between sex and RHR for T2DM development (all P-values were < 0.025 through all models), all analyses were done separately for each gender.

The Cox models’ proportionality was measured using the Schoenfeld residual test. All proportionality assumptions were appropriate. Statistical analyses were employed by the STATA version 14 (Stata Corp LP, College Station, Texas) statistical software. P-values < 0.05 were considered statistically significant.

## Results

The mean age of the male and female participants was 41.5 and 39.1 years, respectively. Baseline characteristics of the male and female participants across quantiles of RHR are presented in Tables 1 and 2, respectively. For continuous variables, there were significant differences in cardiometabolic profile across RHR quantiles among men except for age and HDL-C; however, generally, there was no significant corresponding difference among women. Moreover, generally, in the higher RHR quantiles, the prevalence of low physical activity was increased in both sexes.

**Table 1:** Baseline characteristics according to the resting heart rate quantiles among men: Tehran Lipid and Glucose Study

	Q1	Q2	Q3	Q4	Q5	P-value*
Number of participants	374	590	355	520	592	
Resting heart rate range	< 68	68–74	74–78	78–84	≥ 84	
**Continuous variables, Mean ± SD**						
Age (year)	42.84 ± 14.52	41.46 ± 13.50	42.37 ± 13.99	41.00 ± 14.28	40.53 ± 38.00	0.087
BMI (kg/m^2^)	25.67 ± 3.90	25.91 ± 3.91	26.28 ± 3.88	26.19 ± 4.37	26.54 ± 4.43	0.013
Waist circumference (cm)	91.25 ± 10.30	91.82 ± 10.55	93.14 ± 10.28	92.68 ± 11.39	93.38 ± 11.46	0.014
SBP (mmHg)	113.61 ± 12.56	115.17 ± 14.10	115.10 ± 15.38	116.05 ± 14.06	119.74 ± 15.92	< 0.001
DBP (mmHg)	72.12 ± 9.68	74.13 ± 9.66	73.87 ± 9.84	74.83 ± 10.00	77.50 ± 10.68	< 0.001
FPG (mmol/L)	5.01 ± 0.50	4.99 ± 0.47	5.03 ± 0.49	5.08 ± 0.51	5.01 ± 0.50	0.019
HDL-C (mmol/L)	0.94 ± 0.26	0.92 ± 0.21	0.91 ± 0.22	0.90 ± 0.23	0.92 ± 0.22	0.127
TG (mmol/L)	1.66 ± 0.94	1.81 ± 1.11	1.87 ± 0.98	2.06 ± 1.46	1.90 ± 1.08	< 0.001
**Categorical variables, n (%)**						
Education level, years						0.281
- ≤ 6	77 (20.6%)	99 (16.8%)	84 (23.7%)	98 (18.8%)	103 (17.4%)	
- 6–12	218 (58.3%)	365 (61.9%)	198 (55.8%)	301 (57.9%)	362 (61.1%)	
- > 12	79 (21.1%)	126 (21.3%)	73 (20.5%)	121 (23.3%)	127 (21.5%)	
Current smoker, yes	129 (34.5%)	161 (27.3%)	96 (27.0%)	125 (24.0%)	120 (20.3%)	< 0.001
Low physical activity, yes	208 (55.6%)	344 (58.3%)	229 (64.5%)	309 (59.4%)	405 (68.4%)	< 0.001
Family history of DM, yes	102 (27.3%)	174 (29.5%)	89 (25.1%)	142 (27.3%)	183 (30.9%)	0.324

SD: Standard deviation; BMI: body mass index; SBP: systolic blood pressure; DBP: diastolic blood pressure; FPG: fasting plasma glucose; HDL-C: high density lipoprotein cholesterol; TG: triglycerides; DM: diabetes mellitus.

Values are shown as Mean ± SD and number (%), for continuous and categorical variables, respectively; for TG values are shown as Median (interquartile range).

* The comparison p-value between groups was calculated using ANOVA test for continues variables and chi-square test for categorical variables.

**Table 2:** Baseline characteristics according to the resting heart rate quantiles among Women: Tehran Lipid and Glucose Study

	Q1	Q2	Q3	Q4	Q5	P-value*
Number of participants	550	339	702	598	721	
Resting heart rate range	< 76	76–80	80–86	86–92	≥ 92	
**Continuous variables, Mean ± SD**						
Age (year)	41.17 ± 12.20	40.32 ± 11.67	38.69 ± 12.41	38.04 ± 12.69	38.13 ± 12.18	< 0.001
BMI (kg/m^2^)	28.03 ± 4.54	27.92 ± 4.50	27.33 ± 4.82	27.79 ± 5.09	27.65 ± 5.12	0.087
Waist circumference (cm)	88.40 ± 11.79	87.97 ± 11.81	86.88 ± 12.59	87.76 ± 13.15	87.64 ± 12.69	0.299
SBP (mmHg)	109.57 ± 15.39	109.04 ± 13.58	110.24 ± 14.57	110.47 ± 14.11	110.84 ± 15.80	0.332
DBP (mmHg)	72.00 ± 9.80	71.68 ± 8.96	72.86 ± 9.28	73.16 ± 9.23	72.66 ± 9.88	0.090
FPG (mmol/L)	4.95 ± 0.52	4.93 ± 0.49	4.90 ± 0.50	4.89 ± 0.47	4.90 ± 0.50	0.203
HDL-C (mmol/L)	1.08 ± 0.27	1.10 ± 0.29	1.07 ± 0.29	1.10 ± 0.28	1.09 ± 0.27	0.332
TG (mmol/L)	1.46 ± 1.27	1.55 ± 1.01	1.54 ± 0.90	1.54 ± 0.92	1.57 ± 0.95	0.270
**Categorical variables, n (%)**						
Education level, years						0.754
- ≤ 6	159 (28.9%)	90 (26.5%)	175 (24.9%)	147 (24.6%)	188 (26.1%)	
- 6–12	309 (56.2%)	206 (60.8%)	418 (59.5%)	360 (60.2%)	425 (58.9%)	
- > 12	82 (14.9%)	43 (12.7%)	109 (15.6%)	91 (15.2%)	108 (15.0%)	
Current smoker, yes	25 (4.5%)	9 (2.7%)	21 (3.0%)	12 (2.0%)	15 (2.1%)	0.062
Low physical activity, yes	319 (58.0%)	194 (57.2%)	438 (62.4%)	405 (67.7%)	473 (65.6%)	0.001
Family history of DM, yes	171 (31.1%)	101 (29.8%)	194 (27.6%)	180 (30.1%)	226 (31.3%)	0.590

SD: Standard deviation; BMI: body mass index; SBP: systolic blood pressure; DBP: diastolic blood pressure; FPG: fasting plasma glucose; HDL-C: high density lipoprotein cholesterol; TG: triglycerides; DM: diabetes mellitus.

Values are shown as Mean ± SD and number (%), for continuous and categorical variables, respectively; for TG values are shown as Median (interquartile range).

* The comparison p-value between groups was calculated using ANOVA test for continues variables and chi-square test for categorical variables.

During a median follow-up of 12.2 years (IQR: 11.0-13.3), 313 men and 375 women developed T2DM. Sex-specific multivariable HRs for the association of resting heart rate with incident T2DM is reported in Table 3. Among men, compared to the first quintile (< 68 bpm), those who had RHR of over 78 bpm were at higher age-adjusted risk. After adjustment for age, BMI, WC, educational level, low physical activity, current smoking, prevalent hypertension, family history of diabetes, and TG/HDL-C in model 3, this higher risk remained significant for 5th quintile with the HR of 1.55 (95% CI: 1.06–2.26). Moreover, even after further adjustment for baseline FPG, men with RHR of ≥ 84 bpm (5th quintile) had 69% significantly higher risk with the HR of 1.69 (1.16–2.47). Among men, trend of HRs across quintiles was also significant in all models (all P-values were < 0.05). Furthermore, considering RHR as a continuous variable, an increase of 10 bpm caused 17% significantly higher risk in model 4 [HR: 1.17 (1.05–1.30)]. Among women, on the other hand, there was no significant association between incident T2DM and RHR (both as a categorical or continuous variable).

**Table 3:** Multivariable hazard ratios (HR) and 95% confidence intervals (CI) for the association of resting heart rate with incident type 2 diabetes mellitus: Tehran Lipid and Glucose Study

	Quantile Range (bpm)	E/N	Model 1		Model 2		Model 3		Model 4	
			h (95% CI)	P-value	HR (95% CI)	P-value	HR (95% CI)	P-value	HR (95% CI)	P-value
**Men**										
**First Quantile**	< 68	40/374	Reference		Reference		Reference		Reference	
**Second Quantile**	68–74	64/590	1.11 (0.75–1.65)	0.598	1.08 (0.73–1.61)	0.696	1.08 (0.72–1.60)	0.719	1.15 (0.77–1.72)	0.489
**Third Quantile**	74–78	40/355	1.12 (0.72–1.74)	0.608	1.08 (0.69–1.67)	0.748	1.05 (0.68–1.63)	0.831	1.21 (0.78–1.89)	0.389
**Fourth Quantile**	78–84	76/520	1.56 (1.06–2.29)	0.023	1.49 (1.01–2.19)	0.045	1.43 (0.97–2.11)	0.072	1.33 (0.90–1.96)	0.159
**Fifth Quantile**	≥ 84	93/592	1.76 (1.21–2.54)	0.003	1.57 (1.07–2.30)	0.020	1.55 (1.06–2.26)	0.025	1.69 (1.16–2.47)	0.007
**P-value for trend**				0.004		0.032		0.048		0.049
**Increase of 10 bpm**		313/2431	1.21 (1.09–1.34)	< 0.001	1.17 (1.05–1.30)	0.005	1.16 (1.04–1.30)	0.007	1.17 (1.05–1.30)	0.005
**Women**										
**First Quantile**	< 76	80/550	Reference		Reference		Reference		Reference	
**Second Quantile**	76–80	46/339	0.98 (0.68–1.41)	0.931	0.98 (0.68–1.41)	0.900	0.92 (0.64–1.32)	0.648	1.00 (0.70–1.44)	0.993
**Third Quantile**	80–86	84/702	0.90 (0.66–1.22)	0.502	0.90 (0.66–1.23)	0.514	0.88 (0.65–1.20)	0.418	0.97 (0.71–1.32)	0.838
**Fourth Quantile**	86–92	70/598	0.93 (0.67–1.28)	0.645	0.83 (0.60–1.15)	0.264	0.80 (0.58–1.11)	0.185	0.88 (0.64–1.21)	0.430
**Fifth Quantile**	≥ 92	95/721	1.07 (0.79–1.44)	0.674	0.96 (0.71–1.30)	0.806	0.92 (0.68–1.25)	0.608	0.97 (0.72–1.31)	0.845
**P-value for trend**				0.822		0.813		0.758		0.941
**Increase of 10 bpm**		375/2910	1.02 (0.93–1.12)	0.658	0.98 (0.89–1.08)	0.656	0.97 (0.88–1.07)	0.532	0.98 (0.89–1.08)	0.661

E: event; N: number; bpm: beat per minute.

Model 1: Adjusted for age.

Model 2: adjusted for age, body mass index, waist circumference, educational level, low physical activity, current smoking, prevalent hypertension, and family history of diabetes.

Model 3: Model 2 + further adjusted for Triglycerides/ High-density lipoprotein cholesterol ratio (TG/HDL-C).

Model 4: Model 3 + further adjusted for fasting plasma glucose.

As a sensitivity analysis, after adjustment for HOMA-IR, an increase of 10 bpm had HRs of 1.09 (0.94–1.27, P-value: 0.24) and 1.01 (0.90–1.14, P-value: 0.83) among a subgroup of 1394 men and 1849 women, respectively.

As a secondary analysis, we evaluated the relation of RHR with incident T2DM among prediabetic participants (Table 4). Generally, among male and female participants, the relationship was similar to non-diabetic ones; although there was no significant association among women with prediabetes at baseline, an increase of 10 bpm was associated with 26% significantly higher risk of T2DM development among prediabetic men in model 4 [HR: 1.26 (1.09–1.46)]. Moreover, compared to the prediabetic men with RHR of < 68 bpm, RHR of > 80 bpm showed significant increased risk of incident T2DM in model 4.

**Table 4:** Multivariable hazard ratios (HR) and 95% confidence intervals (CI) for the association of resting heart rate with incident type 2 diabetes mellitus among subjects with pre-diabetes at baseline: Tehran Lipid and Glucose Study

	Quantile Range (bpm)	E/N	Model 1		Model 2		Model 3		Model 4	
			h (95% CI)	P-value	HR (95% CI)	P-value	HR (95% CI)	P-value	HR (95% CI)	P-value
**Men**										
**First Quantile**	< 68	20/73	Reference		Reference		Reference		Reference	
**Second Quantile**	68–75	34/95	1.53 (0.88–2.66)	0.131	1.50 (0.86–2.61)	0.155	1.49 (0.86–2.61)	0.157	1.49 (0.85–2.61)	0.160
**Third Quantile**	75–80	40/118	1.39 (0.81–2.38)	0.231	1.52 (0.89–2.61)	0.129	1.52 (0.88–2.61)	0.133	1.56 (0.91–2.69)	0.107
**Fourth Quantile**	80–84	34/78	1.93 (1.11–3.35)	0.020	1.93 (1.10–3.39)	0.021	1.92 (1.10–3.38)	0.023	1.79 (1.01–3.14)	0.045
**Fifth Quantile**	≥ 84	53/119	2.09 (1.25–3.50)	0.005	2.12 (1.26–3.59)	0.005	2.12 (1.25–3.58)	0.005	2.22 (1.31–3.76)	0.003
**P-value for trend**				0.039		0.055		0.057		0.046
**Increase of 10 bpm**		181/483	1.25 (1.08–1.44)	0.002	1.25 (1.08–1.44)	0.003	1.25 (1.08–1.44)	0.003	1.26 (1.09–1.46)	0.002
**Women**										
**First Quantile**	< 74	30/76	Reference		Reference		Reference		Reference	
**Second Quantile**	74–80	36/90	0.91 (0.56–1.48)	0.706	0.89 (0.55–1.46)	0.652	0.88 (0.54–1.43)	0.600	0.93 (0.57–1.52)	0.772
**Third Quantile**	80–89	59/147	1.04 (0.67–1.61)	0.872	0.99 (0.64–1.54)	0.963	0.97 (0.62–1.51)	0.882	1.09 (0.70–1.70)	0.699
**Fourth Quantile**	89–94	50/102	1.29 (0.82–2.02)	0.277	1.19 (0.76–1.88)	0.451	1.14 (0.72–1.80)	0.584	1.24 (0.78–1.96)	0.367
**Fifth Quantile**	≥ 94	45/112	1.03 (0.65–1.64)	0.899	0.88 (0.55–1.41)	0.598	0.88 (0.55–1.42)	0.609	0.95 (0.59–1.52)	0.824
**P-value for trend**				0.584		0.619		0.741		0.654
**Increase of 10 bpm**		220/527	1.03 (0.91–1.16)	0.480	0.99 (0.88–1.12)	0.884	0.99 (0.88–1.12)	0.870	1.01 (0.90–1.14)	0.859

E: event; N: number; bpm: beat per minute.

Model 1: Adjusted for age.

Model 2: adjusted for age, body mass index, waist circumference, educational level, low physical activity, current smoking, prevalent hypertension, and family history of diabetes.

Model 3: Model 2 + further adjusted for Triglycerides/ High-density lipoprotein cholesterol ratio (TG/HDL-C).

Model 4: Model 3 + further adjusted for fasting plasma glucose.

## Discussion

In this prospective population-based cohort study, during over a decade of follow up, we found a significant interaction between sex and RHR for the T2DM risk. After adjustment for traditional T2DM risk factors, TG/HDL-C ratio, and baseline FPG, an increase of 10 bpm was associated with about 17% higher risk of T2DM development among men. Moreover, compared to RHR of < 68 bpm, men with RHR of ≥ 84 bpm had about 70% increased risk of incident T2DM; however, this higher risk attenuated to an insignificant level after adjustment for HOMA-IR. Among women, on the other hand, there is no association between RHR and T2DM development. Furthermore, the relationship between RHR and T2DM risk among prediabetic men and women was also similar to non-diabetic ones.

Although several studies have been published about the association of RHR and T2DM, their findings are not completely comparable to ours, due to differences in study design, study setting, outcome assessment, level of adjustment, and other methodological aspects. Similar to our findings for men, in a prospective cohort study of 31,156 male health professionals, highest versus lowest categories of RHR had an about 70% increased risk of T2DM development; moreover, an increase of 10 bpm showed 19% higher risk [10]. In a meta-analysis of the mentioned study [10] and another 13 prospective cohort studies, a positive association between RHR and T2DM risk was found; the summary relative risk (RR) per 10 bpm increase was 1.17 (95% CI, 1.09–1.26); moreover, the summary RR for highest versus lowest RHR was reported to be 1.44 (1.20–1.74) in the meta-analysis [10]. Similarly, two other meta-analyses [8, 11] showed higher risk of T2DM for increased range of RHR. Findings of recently published cohort studies from Asian countries were also similar [17–19].

Furthermore, in the current study, we evaluated the relationship of RHR with risk of progression from prediabetes to T2DM; similar to non-diabetic participants, increased RHR caused significantly higher risk of T2DM development only among men. Similarly, in another longitudinal study, it was shown that higher RHR at baseline was associated with a modestly increased incidence rate of T2DM among American overweight adults with prediabetes [20]. Additionally, from a prospective study from China, the researchers have found that fasting RHR was associated with higher risk of progression from impaired fasting glucose to diabetes [11]; however, their findings were significant not only for men but also for women, which was different from our findings [11].

Several different mechanisms were introduced previously for the explanation of the association between increased RHR and risk of T2DM development that mostly attributed to insulin resistance (IR) induction by autonomic system [10]. Since RHR is an indicator of autonomic activity [21], higher RHR indicates a change in the sympathetic-parasympathetic balance in favor of the sympathetic. It causes glucose metabolism dysregulation through: (1) reduced insulin secretion, (2) reduced skeletal muscle glucose uptake by vasoconstriction, and (3) elevated IR in the skeletal muscle cells, by the stimulation of renin-angiotensin-aldosterone system [8, 10, 22, 23]. It should be noted that based on a meta-analysis, it was suggested that baseline glucose and/or IR accounts for part, though not all of the RHR and incident T2DM relation [8]. Recently, it was reported that only 27.5% of the RHR effect on incident T2DM was explained by the indirect effect of IR [24]. Importantly, chronic sympathetic over-activity was linked to high blood pressure, obesity, and the metabolic syndrome that all of them are accompanied by T2DM development, on the basis of high inflammatory state [25]. In our models, even after adjustment for obesity, hypertension, TG/HDL-C ratio and baseline FPG, the association remained significant among men; however, after adjustment for HOMA-IR, this higher risk did not remain significant. Moreover, lower RHR is a potential marker of better cardiorespiratory fitness [26], which can provide protection against cardio-metabolic diseases including T2DM [27]; however, the low-physical-activity, which was adjusted in our models, could not completely evaluate the cardiorespiratory fitness. Finally, evidence of genetic causal correlations between RHR and T2DM/cardiometabolic traits was also found [28]. Nevertheless, further investigations are needed yet to clarify this complex relationship [29].

In contrast to a meta-analysis study [8] and some other previous studies that did not find a significant interaction between RHR and sex on the T2DM risk [11, 18, 30], this interaction was significant in the current study, even after adjustment for age, BMI, WC, educational level, low physical activity, current smoking, prevalent hypertension, family history of diabetes, TG/HDL-C ratio, and FPG. Despite a significant association between RHR and T2DM among men, our female participants did not show any significant difference in T2DM risk across RHR quantiles. Based on a prospective cohort study among Inner Mongolian, a similar significant interaction was also found for sex; however, higher quartile of RHR caused higher T2DM risk in both genders, although it is more prominent among men [19]. Similarly, some other studies also reported this higher impact of RHR on T2DM risk among men [31, 32].

Several physiological differences may explain different findings in males and females. Firstly, sex steroid hormones play an important role in protecting women against T2DM development through enhancing insulin sensitivity by activating estrogen receptor α in insulin sensitive tissues such as skeletal muscles, adipose tissue, and hepatocytes [33, 34]. Moreover, higher mitochondrial activity in different tissues such as adipose tissue and skeletal muscle in female gender caused further protection against T2DM development [34]. Secondly, considering the autonomic nervous system, which regulates RHR, vagal and parasympathetic activity in female heart is more prominent than male [35]. Oxytocin also increases vagal activity and decreases RHR in women [36]. Furthermore, the association between RHR and elevated levels of all the inflammatory markers is further prominent in men than in women [37]. Therefore, in women, RHR may not be as accurate as among men for indication of high inflammatory state and sympathetic-parasympathetic imbalance.

As strengths, this is the first prospective study investigating the impact of RHR on incident T2DM in the MENA region, with a high burden of T2DM. Another strength of our study is adjusting sex-specific models for several potential confounders. We also acknowledge several limitations. First, in the current study, RHR was measured by radial pulse counting over 60-s periods that was less accurate than using electrocardiogram which measures the heart rate directly; this issue may become more important in older age when atherosclerosis is more involved. Second, the present study only included Tehranian citizens; hence, the results may be unable to be generalizable to the other ethnicities or rural populations.

## Conclusion

To sum up, among non-diabetic and prediabetic men, higher RHR was significantly associated with higher risk of incident T2DM. For women, on the other hand, there was no significant relationship. Further studies are needed to determine the added value of RHR as a potential modifiable risk factor in screening and prediction of incident T2DM.

## Data Availability

The datasets used and/or analyzed during the current study are available from the corresponding author on reasonable request.

## Abbreviations

T2DM: Type 2 diabetes mellitus
DALYs: Disability-adjusted life years
MENA: Middle East and North Africa
RHR: Resting heart rate
IR: Insulin resistance
bpm: Beats per minute
TLGS: Tehran Lipid and Glucose Study
NCDs: Non-communicable diseases
CVD: Cardiovascular disease
FPG: Fasting plasma glucose
2h PG: 2-h post-challenge plasma glucose
BMI: Body mass index
BP: Blood pressure
HDL-C: High-density lipoprotein cholesterol
TG: Triglyceride
SBP: Systolic blood pressure
DBP: Diastolic blood pressure
MAQ: Modifiable Activity Questionnaire
GLDs: Glucose lowering drugs
HOMA-IR: Homeostasis Model Assessment of Insulin Resistance
SD: Standard deviation
IQR: Interquartile range
HRs: Hazard ratios
CIs: Confidence intervals
RRs: Relative risk
